# Pattern of physical activity among persons with type 2 diabetes with special consideration to daily routine

**DOI:** 10.12669/pjms.321.8379

**Published:** 2016

**Authors:** Rozina Arshad, Bilal Bin Younis, Junaid Masood, Maham Tahira, Saima Khurhsid

**Affiliations:** 1 Rozina Arshad, Sakina Institute of Diabetes and Endocrine Research, Shalamar Medical & Dental College and Hospital, Lahore, Pakistan; 2 Bilal Bin Younis, Sakina Institute of Diabetes and Endocrine Research, Shalamar Medical & Dental College and Hospital, Lahore, Pakistan; 3 Junaid Masood, Sakina Institute of Diabetes and Endocrine Research, Shalamar Medical & Dental College and Hospital, Lahore, Pakistan; 4 Maham Tahira, Sakina Institute of Diabetes and Endocrine Research, Shalamar Medical & Dental College and Hospital, Lahore, Pakistan; 5 Saima Khurhsid, Sakina Institute of Diabetes and Endocrine Research, Shalamar Medical & Dental College and Hospital, Lahore, Pakistan

**Keywords:** Diabetes, Physical Activity, House Hold Activities

## Abstract

**Objective:**

Physical activity is essential in maintaining a healthy lifestyle. Physical activity can improve general health, quality of life and diabetes management. The aim and objective of the study was to assess the physical activity trends in daily routine of people with type 2 diabetes.

**Methods:**

Two hundred persons with diabetes from four different clinical settings were included to access the trends of physical activity using a customized questionnaire EPIC-2. Pattern of physical activity was assessed across a set of domains including sleep time, hours of TV watch, preferred mode of transport for specific distance and household activities. Data was analyzed using SPSS 21.

**Results:**

Out of 200 persons with diabetes, 104(52%) were male and 96 (48%) were female. Out of the total sample of patients, 85 (81.7%) Male and 80 (83.3%) female patients preferred walk to cover a distance of less than one mile. There was a significant difference in selection of mode of transport for all other specified distance, esp. in female patients with both age groups. There was insignificant difference for physical activity pattern related to household activities in young and elderly male subjects. The mean sleeping time for younger male subjects on weekend was 464.31± 88.88 minutes/day and for elder it was 418.65± 102.66 minutes/day while for young female subjects was 476.25± 113.74 minutes/day and in female elderly subjects it was 420.62± 120.62 minutes/day respectively.

**Conclusion:**

In type 2 diabetics we observed a low level of physical activity which may be detrimental for the control of diabetes mellitus.

## INTRODUCTION

Physical activity is defined as all forms of activity resulting in body movement including exercise, cardiorespiratory fitness and physical fitness. Lack ofPhysical activity is an established risk factor for cancer, cardiovascular disease and diabetes, which along with chronic respiratory disease accounts for more than 60% of all deaths.^1^ The most recent estimates suggest that approximately two million deaths per year worldwide are due to inactivity.^2^ Regardless of global trepidations about non-communicable disease, increasing obesity and rapid changes in arrangements of work, transport and recreation, monitoring of physical activities isbeing carried out insufficiently.^3^,^4^

However, major improbability always remain, in forming the relationship between activity and health outcome, mostly due to the method by which physical activity is measured and reported. Improving the assessment of physical activity in epidemiological studies is considered a challenge, particularly in the rapidly growing population segment comprising older adults, this needs special attention with regards to physical activity and its influence on health. Physical activity can be assessed by subjective methods e.g. questionnaires, activity diaries^5^ and objective methods e.g. motion sensors, heart rate monitors, combined sensor.^6^ Objective method is useful for the intensity of activity but not for type of activity. Questionnaire based assessment of physical activity is the most commonly applied method in epidemiological studies as it has an advantage of ease of administration, low cost and low participant and researcher burden.^7^,^8^ Most of the questionnaires focus on physical activity at work place or during leisure time. Only few questionnaires assess physical activity in a variety of situations including work, transportation, recreation and domestic life.^9^,^10^ The EPIC norfolk physical activity questionnaire (EPAQ2) was designed for the assessment of physical activity in the Norfolk cohort of the European prospective investigation into cancers.^11^ Studies showed that several socio-economic aspects are associated with physical inactivity, for example professionals and skilled workers were more inactive than unskilled workers in the specific region.^12^ Likewise higher education was a significant factor associated with physical inactivity.^13^ Low physical activity is also one of the contributing risk factors for the higher obesity levels seen among Asian Indians.^14^ Among South Indians, Vaz et al. reported that individuals engaging in recreational exercise were inactive in other domains.^15^,^16^

The objective of this study was to assess the physical activity trends in daily routine of people with diabetes on the basis of gender and age.

## METHODS

It was a cross sectional and observational study. In this study, the EPIC Norfolk physical activity questionnaire (EPAQ2) customized was used to gauge 200 persons with type 2 diabetes. The participants of the study from Shalamar Hospital, Lahore and three different premises of Lahore, were interviewed on specific domains for example hours of TV watch, sleep time during weekdays and at weekends, hours of TV watch, preferred mode of transport for specific distance, household activities which further includes time allocated to cooking, shopping, ironing, laundry, care of elders. The physical activity questionnaire was filled in by a trained nurse and diabetes educator. Informed consent of the patients participating in the study were undertaken.

The study protocol was approved by the Institution Review Boards of the Shalamar Hospital and other premises. Chi square test was applied and p<0.05 is considered as statistically significant. Statistical Package for Social Sciences version 21(SPSS) Chicago, IL, USA was used for statistical analysis.

## RESULTS

Two hundred people with diabetes were enrolled in this study from different diabetic clinics at Lahore, Pakistan. The mean age of the sample population was 44.70± 11.90 years of which 104(52%) were male and 96(48%) were female subjects as shown in the Table-I. The four means that were noted for travel. The majority of male subjects 85(81.7%) choose walk to cover a distance less than one mile, while 80(83.3%) female prefer to walk for this distance. More than one uptil five miles distance was covered by almost all means of transport like 18(17.3%) walked, 40(38.5%) cycle, 23(22.1%) car and 23(22.1%) on public transport by male whereas for female the choice to cover same distance was 18(18.8%) by walked, 3(3.1%) by cycle, 38(39.6%) by car and 13(13.5%) on public transport. For male subjects, the preferred mode to travel for more than five miles was public transport 63(60.6%) followed by car in 30(28.8%) while for female the numbers was 51(53.1%) by car, followed by 45(46.9%)by public transport. There was significant difference in selection of mode of transport for all three specified distance for patients with age group <40 years and >40 years for female patients. These are depicted in Table-II and Table-III.

**Table-I:** Characteristics of patients.

Characteristics	Number
Total population	200
Mean age(years) M±SD	44.7±11.09
Male	104(52%)
≤ 40 years	44(42.3%)
> 40 years	60(57.7%)
Female	96(48%)
≤ 40 years	24(25%)
> 40 years	72(75%)

Data are represented as n (%) and M±SD where applicable.

**Table-II:** Preferred mode to cover a distance (male).

	Walk	Cycle	Car	Public Transport	P value
< 1 mile	85(81.7)	87.7)	8(7.7)	3(2.9)	0.052
<40 year	32(30.8)	7(6.7)	4(3.8)	1(1)	
>40year	53(51)	1(1)	4(3.8)	2(1.9)	
1-5 miles	18(17.3)	40(38.5)	23(22.1)	23(22.1)	
<40 year	1(1)	29(27.9)	7(6.7)	7(6.7)	<0.001*
>40year	17(16.3)	11(10.6)	16(15.4)	16(15.4)	
>5 miles	0(0)	11(10.6)	30(28.8)	63(60.6)	
<40 year	0(0)	6(5.8)	8(7.7)	30(28.8)	0.110
>40year	0(0)	5(4.8)	22(21.2)	33(31.7)	

Data are represented as n (%). Numbers might not add to 100% because of rounding, Chi Square test used.

**Table-III:** Preferred mode to cover a distance (female).

	Walk	Cycle	Car	Public Transport	P value
< 1 mile	80(83.3)	0(0)	14(14.6)	2(2.1)	
<40 year	19(19.8)	0(0)	3(3.1)	2(2.1)	0.046*
>40year	61(63.5)	0(0)	11(11.5)	0(0)	
1-5 miles	23(24)	9(9.4)	42(43.8)	22(22.9)	
<40 year	5(5.2)	6(6.3)	4(4.2)	9(9.4)	0.001*
>40year	18(18.8)	3(3.1)	38(39.6)	13(13.5)	
>5 miles	0(0)	0(0)	51(53.1)	45(46.9)	
<40 year	0(0)	0(0)	8(8.3)	16(16.7)	0.025*
>40year	0(0)	0(0)	43(44.8)	29(30.2)	

Data are represented as n (%). Numbers might not add to 100% because of rounding, Chi Square test used.

The TV watching among male and female of less than 40 years and more than 40 years during weekday and weekends were noted. The majority of the male subjects 69(66.3%) were not in habit of watching TV before 6pm on a weekday and 33(31.7%) use to watch TV <1 hour/day after 6pm while 62(64.4%) female subjects reported that they were not in habit to watch TV on weekdays before 6pm. On weekend, 47(45.2%) male did not watch TV before 6pm and 25(24%) use to watch TV 1 to 2 hours/day after 6pm. There was significant difference for male in habit of watching TV before 6pm on weekday, before 6pm on weekend and after 6pm on weekend in young and elderly patient.

Other type of household physical activities were also noted. There was a large number of patients who did not perform any type of physical activity. Among male, 85(81.7%) did not spend their time on preparing food or washing up, 76(73.1%) did not go for shopping and browsing, 95(91.3%) were not involved in cleaning house, 98(94.2%) responded negatively when asked about ironing and laundry, and 79(76%) were not involved in care of handicapped elderly people. There was insignificant difference for physical activity pattern in young and elderly patients.

Among female 24(25%) did not spend their time on preparing food or washing, 34(35.4%) did not go for shopping of grocerries, 41(42.7%) did not go for shopping and browsing of other than grocery items 50(52.1%) were not involved in cleaning house, 77(80.2%) responded negatively when asked about ironing and laundry, and 94(97.9%) were not involved in care of handicapped elderly people. There was significant difference for physical activity pattern in young and elderly patients for shopping and browsing, cleaning house, ironing and laundry, care of handicapped elderly people activities.

The mean time to sleep in young female subjects was 423.75±77.28 minutes/day and in female elderly subjects it was 413.12±95.69 minutes/day on weekdays while on weekends it was 476.25± 113.74minutes/day and 420.62± 120.62 minutes/day respectively. On the other hand, the mean sleeping time for younger male subjects on weekend was 464.31± 88.88 minutes/day and for elder it was 418.65±102.66 minutes/day as depicted in Table-IV.

**Table-IV:** Sleep time on weekends and weekdays.

	Sleep time on weekdays (mins/day)	Sleep time on weekends(mins/day)
*Female*		
<40	423.75± 77.28	476.25± 113.74
>40	413.12± 95.69	420.62±120.62
*Male*		
<40	442.5 ± 58.98	464.31± 88.88
>40	405 ± 85.83	418.65±102.66

Data are represented as n (%) and M±SD where applicable.

## DISCUSSION

The present study mainly focuses on the prevalence of different forms of physical activity and its variations with gender and age in people with type 2 diabetes. We also tried to identify past time leisure activities and in different domains like work, transport, occupations. According to our study younger population were more active than elder ones, irrespective of gender and males are more physically active than females irrespective of age. The results of walking activity are comparable to the results of a qualitative study conducted among South Asians living in United Kingdom showing the pattern of walking in Pakistani males,^17^ this study showed that out of 156 Pakistani males 93(59.6%) were walking < 1 mile, 48 males (30.1%) walked 1-3 miles on an average weekday and 15 Pakistani males (9.6%) were involved in walking > 4 miles on a weekday. where as in our study none covered >5miles distance by walking. As far as cycling is concerned our results are in contrast to the results of the study conducted at UK, in which 100% of Pakistani males cycled <2 miles on weekdays and none were involved in cycling of >2miles. One possible explanation to results of our study regarding use of different mode of transportation and using cycling as preferred mode of transportation, may be due to financial constraints and limited availability of the public transport. Cycling by women as a mode of transportation is very low as this is not a culturally acceptable norm. The activity undertaken by females in our study is mainly reported as home activities. Social and Cultural values may limit the involvement of women in certain types of physical activity in some religious and ethnic groups. The customary part of Pakistani women in taking care of domiciliary work and supporting extended family members may limit the time available for them to engage in particularly leisure time physical activities.^17^

We observed that female subjects are more involved in shopping, cooking, ironing, laundry and caring of their husbands and preschool children. Our study results are not very different compared to the results of the study conducted in UK in which 137(96.5%) of Pakistani females did not cycled at all and 1.4%^2^ females cycled <2-6 miles on weekdays The Health Survey for England reported low levels of heavy manual activities among Indian (19 per cent), Pakistani (12 per cent) and Bangladeshi (5 per cent) men compared to men in the general population (30 per cent). Fast or brisk walking was also less commonly reported. Only 5, 5 and 3 percent of Indian, Pakistani and Bangladeshi women respectively, reported heavy manual work, compared to 12 per cent of women from the general population.^18^ Pomerleau et al.reported that ‘South Asian’ women were less likely to cycle or take part in sports, although they were more physically active at work.^19^

The time spent in TV watching in both male and female was also observed. It is in some way reflected the results shown by Dunstan and associates in which increased blood glucose level with increased TV watching.^20^

We have used a validated questionnaire, only few studies reported validation of the physical activity questionnaire. As from our experience of using a validated physical activity questionnaire, it was difficult to extract answers from the sample mainly because of low literacy rate, In the light of our study we strongly recommend exercise an physical activity should be promoted through public campaign however it is important to understand socioeconomic factors associated with different population in order to deliver effective physical activity intervention.

## CONCLUSION

There is difference in type of physical activities in both gender perhaps due to our social norms and expectation of being subservient from females. Time constraints lack of awareness and busy life might have lead to suboptimal physical activity. Low levels of physical activity may contribute to the increased risk of diabetes and CHD through effects on obesity and insulin resistance.

### Recommendation

It is strongly recommend that exercise and physical activity should be promoted through public campaign.
